# Zebrafish BID Exerts an Antibacterial Role by Negatively Regulating p53, but in a Caspase-8-Independent Manner

**DOI:** 10.3389/fimmu.2021.707426

**Published:** 2021-08-31

**Authors:** Zhitao Qi, Dong Yan, Lu Cao, Yang Xu, Mingxian Chang

**Affiliations:** ^1^Jiangsu Key Laboratory of Biochemistry and Biotechnology of Marine Wetland, Yancheng Institute of Technology, Yancheng, China; ^2^State Key Laboratory of Freshwater Ecology and Biotechnology, Institute of Hydrobiology, Chinese Academy of Sciences, Wuhan, China; ^3^College of Biotechnology, Jiangsu University of Science and Technology, Zhenjiang, China; ^4^College of Advanced Agricultural Sciences, University of Chinese Academy of Sciences, Beijing, China; ^5^Innovation Academy for Seed Design, Chinese Academy of Sciences, Wuhan, China

**Keywords:** BID, apoptosis, antibacterial activity, p53, *Edwardsiella ictaluri*

## Abstract

Bid (BH3-interacting domain death agonist), a member of the Bcl-2 family, plays a crucial role in the initiation of apoptosis. Independent of its apoptotic function, Bid is also involved in the regulation of inflammation and innate immunity. However, the role of Bid during bacterial pathogen infection remains unclear. In the present study, *Bid* of zebrafish (*Dario rerio*) was cloned and its functions during *Edwardsiella ictaluri* infection were investigated. Zebrafish Bid enhances the apoptosis rate of *Epithelioma papulosum cyprini* (EPC) cells following *E. ictaluri* infection. Importantly, *in vitro* and *in vivo* bacterial invasion assays showed that overexpressed Bid could significantly inhibit the invasion and proliferation of *E. ictaluri*. Real-time qPCR analysis revealed that p53 gene expression was downregulated in embryos microinjected with Bid-FLAG. Further, *in vitro* and *in vivo* bacterial invasion assays showed that overexpressed p53 increased the invasion and proliferation of *E. ictaluri*. Moreover, the invasion and proliferation of *E. ictaluri* were inhibited when co-overexpressing Bid and p53 *in vivo* and *in vitro*. Further, the numbers of *E. ictaluri* in larvae treated with Z-IETD-FMK (caspase-8 inhibitor) were higher than those of larvae without Z-IETD-FMK treatment, while the number of *E. ictaluri* in larvae microinjected with *bid*-Flag decreased significantly, even if the larvae were treated in advance with Z-IETD-FMK. Collectively, our study demonstrated a novel antibacterial activity of fish Bid, providing evidence for understanding the function of apoptosis associated gene in pathogen infection.

## Introduction

Apoptosis has crucial roles in biological processes ranging from embryogenesis to aging, and from normal tissue homeostasis to many human diseases (1, 2). The Bcl-2 (B cell lymphoma-2) family is a class of proteins involved in apoptosis. According to their structures and functions, the members of the Bcl-2 family can be divided into three categories, including the multi-domain anti-apoptotic proteins, the multi-domain pro-apoptotic proteins, and the pro-apoptotic BH-3-only proteins (3). The multi-domain anti-apoptotic proteins, including Bcl-2, Bcl-xL, Mcl-1, A1, Bcl-B, and Bcl-w, contain four conserved Bcl-2 homology (BH) domains (BH1-4) and are important for cell survival. The multi-domain pro-apoptotic proteins, Bax, Bak, and Bok, contain BH1-4 domains. The pro-apoptotic BH-3 only proteins include Bid, Bad, Bmf, Bik, Puma, Noxa, Hrk, and Bnip3. By interacting with each other through molecular motifs such as the BH domains, in addition to apoptosis, the Bcl-2 family members are also involved in immune regulation and host immune response to pathogens (4).

Bid (BH3-interacting domain death agonist), a pro-apoptotic protein of the Bcl-2 family, plays a crucial role in the initiation of apoptosis (5). In most cases, Bid is found in the cytoplasm in an inactive state. Under apoptotic stimuli, Bid can be a substrate for a series of proteases, including caspase-8, Granzyme B, calpains, and cathepsins, which generate a truncated Bid (tBid). The tBid then migrates to mitochondria and induces the Bax/Bak-dependent permeabilization of the outer mitochondrial membrane (OM), leading to apoptosis initiation (5, 6). In addition to its role in apoptosis, full-length Bid was found to play crucial roles in inflammation and innate immunity. Full-length Bid could interact with nucleotide-binding and oligomerization (NOD) 1, NOD2, and the IκB kinase (IKK) complex. The cytokine production in colonocytes depleted of Bid or macrophages from *Bid*-/- mice were markedly defective in response to NOD activation (7).

Teleost fish are important animal models for scientific research (8), and Bid is conserved in fish species (9). However, the understanding on the functions of fish Bid is limited. Zebrafish Bid was found to induce apoptosis in embryos (1). Rare minnow (*Gobiocypris rarus*) Bid is involved in grass carp reovirus (GCRV)-triggered apoptosis (10). These results indicated that Bid had conserved pro-apoptotic activity in fish. Interestingly, channel catfish Bid was found to be significantly upregulated at 48 h following *Flavobacterium columnare* infection (11), giving evidence that fish Bid may play some function during bacterial invasion. Further research is needed to elucidate the function and mechanism of fish Bid during bacterial infection.

*Edwardsiella ictaluri*, a Gram-negative bacteria, is a deadly pathogen in zebrafish (*Danio rerio*) (12, 13). In the present study, we found that zebrafish Bid could induce apoptosis in piscine cells, similar to previous observations in mammals (1, 14). Furthermore, zebrafish Bid exhibited antibacterial activity, which could significantly inhibit the invasion and proliferation of *E. ictaluri in vivo* and *in vitro*. More importantly, we found that the antibacterial activity of zebrafish Bid works in a caspase-8 independent fashion. This is the first report about the antibacterial activity of fish Bid. Our study enriches the knowledge about the function of fish Bid, which could be used as a potential treatment choice for bacterial infection in fish.

## Materials and Methods

### Cell and Bacteria

*Epithelioma papulosum cyprinid* (EPC) cells were cultured in an M199 (HyClone, USA) culture medium with 10% fetal bovine serum (FBS) (Lonsa, USA). Wild-type *E. ictaluri* (PPD130/91 strain) were cultured in tryptic soy broth (TSB, BD Biosciences) at 28°C.

### Plasmid Construction and Sequence Analysis

The specific primers (bid-F1: CCGGAATTCATGGACTTCAACAGGAAC and bid-R1: were designed according to the zebrafish Bid EST sequence (GenBank accession No: XM_005159109.4) and used for RT-PCR to clone the full open reading frame (ORF) of zebrafish Bid with the cDNA of the wild type of zebrafish as templates. The PCR products and p3xFLAG-CMV™-14 were digested using EcoR I and Xba I restriction enzymes and ligated using T4 DNA ligase (TaKaRa, USA). All plasmids were confirmed by sequencing.

### Expression Analysis of Bid

To investigate the expression changes of zebrafish Bid during early development, wild-type zebrafish embryos or larvae were collected at 6 h, 12 h post-fertilization (hpf), 1 day (d), 3 days, 5 days, and 10 days post-fertilization (dpf). For each time point, 30–50 embryos or larvae were collected and used for RNA extraction.

To study the inducible expression pattern of Bid, zebrafish larvae at 4 dpf were infected with 2 × 10^8^ CFU/ml *E. ictaluri* in a total volume of 5 ml. After immersion in the bacterial suspension for 6 h, zebrafish larvae were maintained in 60-mm sterile disposable Petri dishes with 25 ml water. Larvae were collected at 12, 24, and 48 h post-infection (hpi). All samples were snap-frozen in liquid nitrogen and stored at −80°C.

### Apoptotic Activity of Zebrafish Bid

To determine the role of Bid in apoptosis, EPC cells seeded overnight in six-well plates at 1 × 10^6^ cells per well were transiently transfected using Lipofectamine 2000 (Invitrogen, USA) with 2 μg p3xFLAG or Bid-FLAG plasmid. At 24 h post-transfection, the cells were infected with various concentrations of *E. ictaluri* with an MOI of 1°C, 5°C, and 10 at 28°C. After infection for 1 h, the medium was removed and replaced with MEM medium containing gentamicin (50 µg/ml) to kill the extracellular bacteria. At 6 hpi, the culture medium was discarded and trypsin without EDTA was added for 2 min. The cell suspension was collected, centrifuged at 4°C and 500 g, and washed twice with precooled PBS solution. Apoptosis was detected using an Annexin V-FITC/PI Apoptosis Detection Kit (Vazyme, China) on a CytoFLEX LX flow cytometer (Beckman, USA).

### Antibacterial Analysis of Zebrafish Bid

For *in vitro* bacterial invasion assays, EPC cells seeded overnight in 24-well plates at 3 × 10^6^ cells per well were transiently transfected using Lipofectamine 2000 (Invitrogen, USA) with 500 ng p3×FLAG or Bid-FLAG plasmid. Twenty-four hours post-transfection, the cells were infected with *E. ictaluri* at MOI 10. After infection for 1.5 h, the medium was removed and replaced with MEM medium containing gentamicin (50 µg/ml) to kill the extracellular bacteria. At 3 and 6 hpi, the cells were washed and lysed in PBS containing 1% (v/v) Triton X-100 at room temperature for 20 min. The lysates were diluted with TBS and then plated onto TSA to calculate the intracellular bacterial colony-forming units (CFU). The invasion assays were performed in triplicate.

For *in vivo* bacterial invasion assays, the p3×FLAG empty plasmid or Bid-FLAG plasmid was diluted to the desired concentration of 200 ng/μl and microinjected into fertilized eggs at the one-cell or two-cell stage with a injected volume of 2 nl. The injected embryos were raised to 4 dpf at 28°C and then were divided into two groups (three replicates per group, with 10 larvae per replicate) used for *E. ictaluri* infection. Briefly, the larvae were infected with *E. ictaluri* at an MOI of 10 for 6 h, then 20 ml aquaculture water was added. At 48 hpi, 10 larvae per group were collected, rinsed with 1 ml PBS, and ground *via* a glass homogenizer to obtain homogenates. Serial dilutions of the homogenates were plated onto TSB agar, and CFU were enumerated after 24 h of incubation at 28°C.

### Expression Analysis of Apoptosis-Related Genes After *Bid* Overexpression

The p3×FLAG empty plasmid or Bid-FLAG plasmid was diluted to the desired concentration of 200 ng/μl and microinjected into fertilized eggs at the one-cell or two-cell stage with an injected volume of 2 nl. The injected embryos were raised to 4 dpf at 28°C, and the apoptosis-related genes, including *birc5*, epidermal growth factor receptor (*egfr*), *caspase 8*, *caspase 3a*, *caspase 3b*, *caspase 9*, programmed cell death protein 8 (*PDCD8*), *bax*, and *bid*, were examined using quantitative real-time PCR (qPCR). The expression of these genes at 12 h post-*E. ictaluri* infection was also examined using qPCR. The bacterial infection was completed as mentioned above. All primers used for gene expressions were listed in **Table S1**.

### Effects of Bid and p53 for Fish Survival and Antibacterial Activity

To further analyze the roles of Bid and p53 for fish survival and antibacterial activity, the fertilized eggs at the one-cell or two-cell stage were randomly divided into three groups (three replicates per group, with 30 eggs per replicate): the control group, which was microinjected with p3×FLAG empty plasmid; the p53 group, which was injected with p53-FLAG; and the p53-Bid group, which was co-injected with Bid-FLAG and p53-FLAG. The injected embryos were raised to 4 dpf at 28°C and then were infected with *E. ictaluri* at an MOI of 10 for 6 days. The numbers of surviving larvae were counted daily, and the survival curves were generated by GraphPad Prism 7. The numbers of *E. ictaluri* in larvae were examined at 24 hpi as the *in vivo* bacterial invasion assays.

### Influence of a Caspase-8 Inhibitor on the Antibacterial Ability of Bid

The fertilized eggs at the one-cell or two-cell stage were pretreated with 50 μM Z-IETD-FMK (caspase-8 inhibitor) (15) for 8 h, then infected with 2 × 10^8^ CFU/ml *E. ictaluri*. The survival rates of larvae were counted for 6 days. The larvae were also collected at 6, 24, and 48 hpi, and the number of *E. ictaluri* in larvae was examined as an *in vivo* bacterial invasion assay. In addition, the 4-dpf larvae, developed from the one-cell or two-cell stage fertilized eggs that were microinjected with p3×FLAG empty plasmid or Bid-FLAG plasmid, were treated with 50 μM Z-IETD-FMK for 8 h and then infected with 2 × 10^8^ CFU/ml *E. ictaluri*. The numbers of *E. ictaluri* in larvae at 24 and 48 hpi were calculated as mentioned above.

### RNA Extraction, cDNA Synthesis, and qPCR

Total RNA was extracted from 30–50 zebrafish embryos or larvae using TRIzol reagent (Invitrogen, USA). The cDNA was reverse transcribed using a RevertAid™ First Strand cDNA Synthesis Kit (Thermo Fisher, USA). The qPCR was performed on a Thermo Fisher Scientific QuantStudio™ 3 Real-Time PCR Instrument (96-well 0.2 ml Block) under the following conditions: 3 min at 95°C, followed by 50 cycles of 15 s at 94°C, 15 s at 52°C–60°C, and 30 s at 72°C. All reactions were performed in triplicate in a 96-well plate, and the mean values were recorded. The specificity of primers was confirmed by sequencing and melting curves. The relative expression of target genes was normalized to the expression of housekeeping genes *gapdh* and *EF-1α* and expressed as fold changes relative to the corresponding control group. The primers used for qRT-PCR are listed in **Table S1**.

### Statistical Analysis

Statistical analysis and graphs were performed and produced using GraphPad Prism 7.0 software. Expression data of qPCR are presented as means and standard error of the mean (SEM). Two-tailed Student’s t-test or ANOVA was used to compare means and SEM between groups. All data are representative of two or three biologic replications. The level of significance is shown as follows: *p < 0.05, **p < 0.01. Significance testing in the cumulative survival analysis used the log-rank test.

## Results

### Sequence Features of Zebrafish Bid

**Figure 1:**
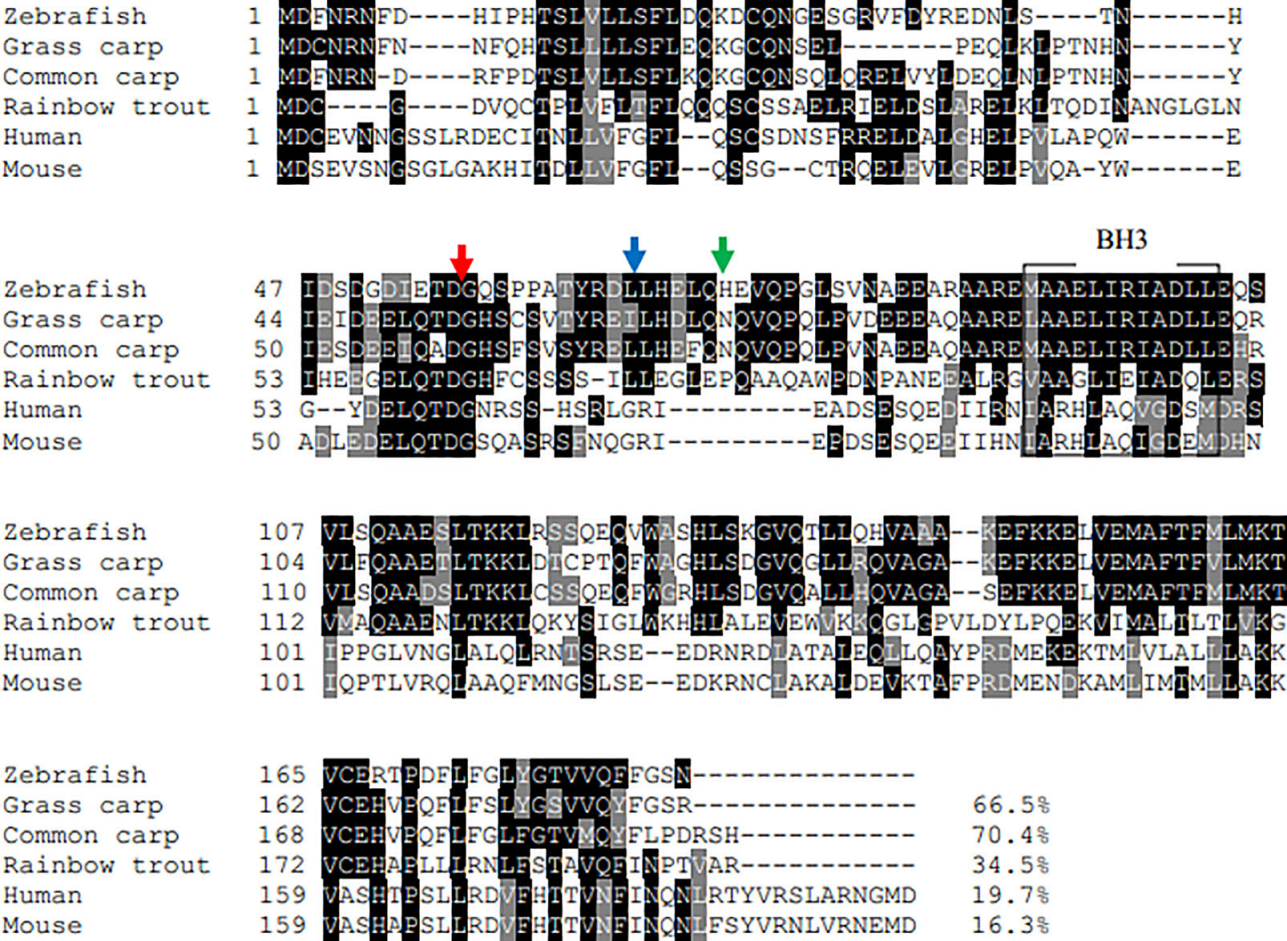
Multiple-sequence alignment of vertebrate Bid. The Bid from each species were aligned using the Clustal O program and decorated with BoxShade software. The conserved BH3 domains were boxed. The caspase-8 cleavage site (LETD), the granzyme B cleavage site (HELQ), and the calpain cleavage site was marked by red arrow, blue arrow, and green arrow, respectively.

**Figure 2:**
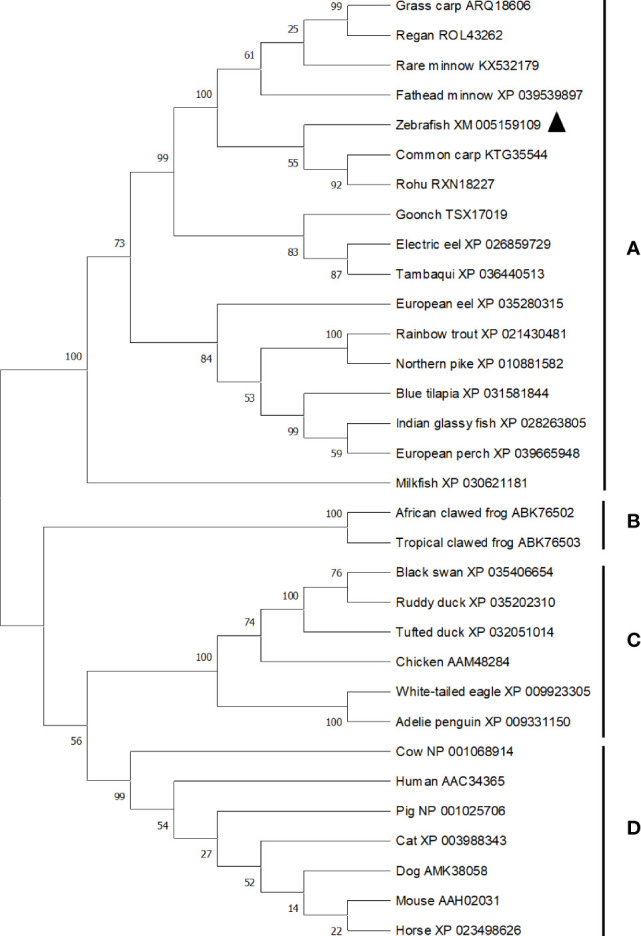
Phylogenetic tree analysis of vertebrates Bid. The phylogenetic tree was constructed using Mega X software using N-J method. Bootstrap was calculated with 10,000 repeats. The tree is divided into four main clades (clade **A–D**), with clade **(A)** composed of fish Bid, clade **(B)** composed of amphibian Bid, clade **(C)** composed of birds Bid and clade **(D)** composed of mammalian Bid. Accession numbers for sequences were listed following the common names of species. Zebrafish Bid was marked by triangle in the tree.

The ORF length of zebrafish *bid* is 564 bp (GenBank accession No. XM_005159109), encoding 187 amino acids. Zebrafish Bid contains a conserved BH3 domain and several conserved cleavage sites (9), including the caspase-8 cleavage site (LETD), the granzyme B cleavage site (HELQ), and the calpain cleave site (**Figure 1**). Zebrafish Bid shares 70.4%, 66.5%, 34.5%, 16.3%, and 19.7% overall sequence identities with the Bid of common carp, grass carp, rainbow trout, mouse, and human, respectively (**Figure 1**). To further understand the evolutionary history of vertebrates’ Bid, a phylogenetic tree was constructed using Mega X software with the neighbor-joining (N-J) method (16). The tree is divided into four main clades (clades A to D), supported by high bootstrap values (≥ 99%) (**Figure 2**). Clade A is composed of Bid of fish. In clade A, zebrafish Bid clustered with the Bid of Cyprinidae. Clade B is composed of Bid from amphibians, and clade C is composed of Bid from birds. Clade D includes the Bid of mammals.

### Expression Analysis of Zebrafish *Bid* in Early Developmental Stages and Larvae Infected With *E. ictaluri*

**Figure 3:**
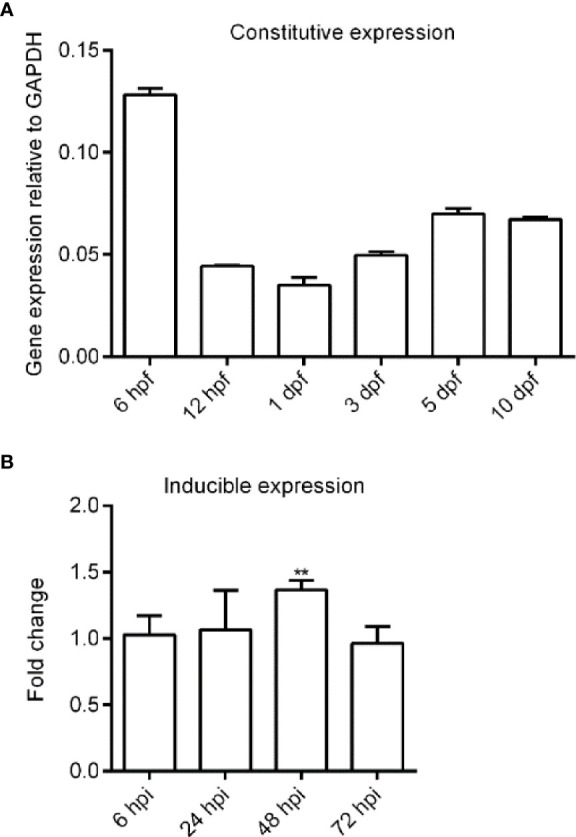
Expression analysis of zebrafish *bid* in normal embryos and embryos infected with *E*. *ictaluri* by using qPCR. **(A)** Expression of *bid* in normal embryos. Thirty to 50 embryos were collected for each time point. Expression of *bid* was normalized to the housekeeping gene *gapdh.* **(B)** Expression of *bid* in embryos infected with *E*. *ictaluri.* The zebrafish larvae at 4 dpf were infected with 2 × 10^8^ CFU/ml *E*. *ictaluri* and were collected at 12, 24, and 48 h postinfection (hpi). The fold changes of *bid* in embryos infected with *E*. *ictaluri* at each time point were analyzed using the 2^-ΔΔCT^ method relative to the corresponding control group. Asterisks indicated that the expression level of *bid* in embryos infected with *E*. *ictaluri* was significantly higher than that of the control. **p < 0.01.

First, we analyzed the expression of zebrafish *bid* by qPCR. Results showed that zebrafish *bid* was expressed ubiquitously across all early developmental stages examined, showing a tendency first to decrease and then increase slowly (**Figure 3A**). We also assessed the expression changes of zebrafish *bid* in larvae infected with *E. ictaluri*. Results showed that zebrafish Bid was significantly upregulated at 48 hpi following *E. ictaluri* infection (**Figure 3B**).

### Apoptotic Activity of Zebrafish Bid

**Figure 4:**
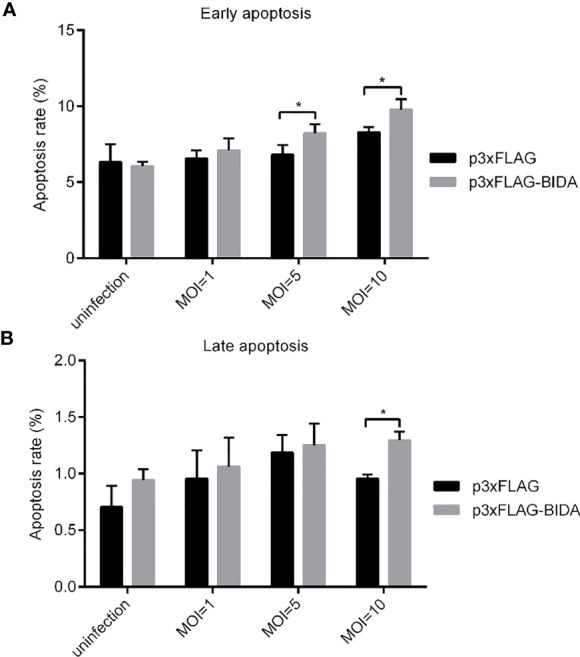
Apoptotic activity of zebrafish Bid at the early apoptosis stage **(A)** and the later apoptosis stage **(B)**. EPC cells were transiently transfected with a 2 μg p3xFLAG or Bid-FLAG plasmid. At 24 hours post-transfection, the cells were infected with E. ictaluri with an MOI of 1, 5, and 10 at 28°C. After infection for 1 h, the extracellular bacteria were killed using gentamicin (50 µg/mL). Apoptosis at 6 hpi was detected using an Annexin V-FITC/PI Apoptosis Detection Kit on the CytoFLEX LX flow cytometer. *p< 0.05.

To test whether Bid can induce apoptosis, we first transfected the EPC cells with Bid-FLAG or p3×FLAG empty plasmid. Then, the cells were infected with *E. ictaluri*, and the apoptosis rates were examined. At the early apoptosis stage, the apoptosis rates for cells transfected with Bid-FLAG were significantly higher than that transfected with empty plasmid when the MOIs of *E. ictaluri* were 5 and 10 (*p* < 0.05) (**Figure 4A**). At the late apoptosis stage, higher apoptosis rates for cells transfected with Bid-FLAG were also observed when the MOI of *E. ictaluri* was 10, compared with those for cells transfected with empty plasmid (**Figure 4B**).

### Antibacterial Activity of Zebrafish Bid

**Figure 5:**
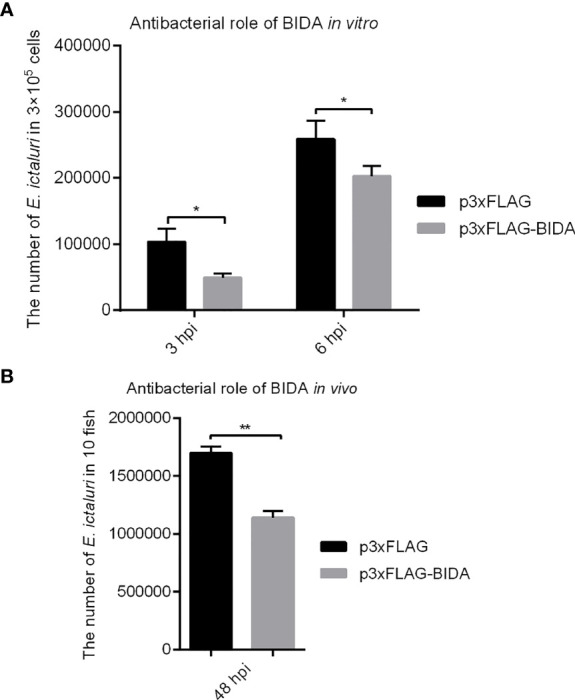
Antibacterial activity of zebrafish Bid *in vitro* **(A)** and *in vivo* **(B)**. For *in vitro* bacterial invasion assays **(A)**, the EPC cells were transiently transfected with 500 ng p3×FLAG or Bid-FLAG plasmid. At 24 h post-transfection, the cells were infected with *E. ictaluri* at an MOI of 10. At 1.5 h post-infection, the extracellular bacteria were killed using gentamicin (50 µg/ml). At 3 and 6 hpi, the cells were lysed, and the intracellular bacterial colony-forming units (CFU) were calculated. For *in vivo* bacterial invasion assays **(B)**, the fertilized eggs were microinjected with 200 ng/μl p3×FLAG or Bid-FLAG. At 4 dpf, the larvae were infected with *E*. *ictaluri* at an MOI of 10. At 48 hpi, the fish were ground, and the CFU was calculated. *p < 0.05, **p < 0.01.

The antibacterial activities of zebrafish Bid were assessed using bacterial invasion assays *in vitro* and *in vivo*. Results showed that overexpression of zebrafish Bid could significantly inhibit the proliferation of *E. ictaluri* in EPC cells at 3 and 6 hpi (**Figure 5A**). *In vivo* bacterial invasion assays showed that the numbers of *E. ictaluri* in fish microinjected with Bid-FLAG were significantly lower than those in fish microinjected with empty plasmid at 48 hpi (**Figure 5B**).

### Zebrafish Bid Inhibits the Transcript of p53

**Figure 6:**
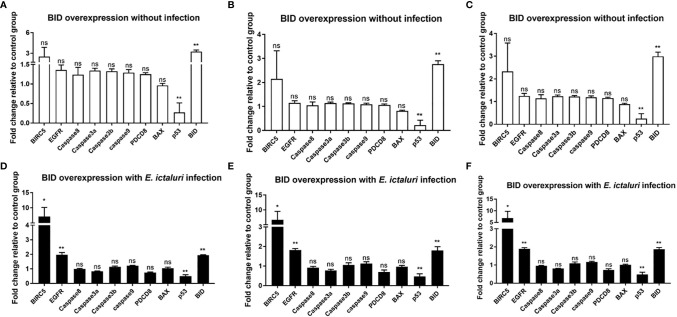
Expression analysis of apoptosis-related genes in *bid*-overexpressed embryos without **(A–C)** or with **(D–F)** *E. ictaluri* infection. The fertilized eggs were microinjected with 200 ng/μl p3×FLAG empty plasmid or Bid-FLAG plasmid. The microinjected eggs were raised to 4 dpf, and the expressions of apoptosis-related genes were examined using qPCR. The 4-dpf microinjected larvae were infected with *E. ictaluri*, and the expressions of apoptosis-related genes were examined using qPCR at 12 hpi. The expression levels of target genes were analyzed using the 2^-ΔΔCT^ method by respectively calculating with the Ct values of *gapdh* **(A, D)**, *EF-1α* **(B, E)**, and the average Ct of *gapdh* and *EF-1α* **(C, F)**. The average Ct values of the two reference genes are listed in **Table S2**. *p < 0.05, **p < 0.01. ns, no significance.

To further understand the antibacterial mechanism of zebrafish Bid, we first examined the expression of *bicr5*, *egfr*, *p53*, *caspase8*, *caspase3a*, *caspase3b*, *caspase9*, *pdcd8*, and *bax* in larvae microinjected with *bid*-FLAG. Results showed that without *E. ictaluri* infection, the expression of p53 was significantly inhibited in larvae microinjected with Bid. No significant changes were observed for other examined genes (**Figures 6A–C**). The larvae microinjected with Bid-FLAG were then infected with *E. ictaluri*, and the expressions of the above genes were examined at 12 hpi. Results showed that the expression of p53 was significantly downregulated and that *birc5* and *egfr* were significantly upregulated (**Figures 6D–F**), when compared with those in larvae microinjected with the p3×FLAG empty plasmid.

### Bid Could Inhibit p53 Activity

**Figure 7:**
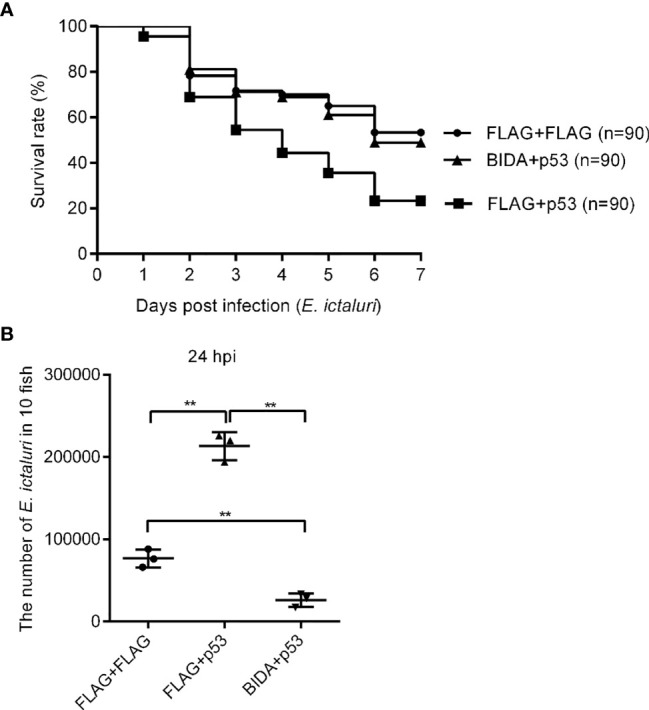
The effects of Bid and p53 on fish survival **(A)** and the number of *E*. *ictaluri* in larvae **(B)**. For fish survival analysis, the fertilized eggs were microinjected with p3×FLAG empty plasmid, p53-Flag, or co-microinjected p53-Flag and *bid*-Flag and raised to 4 dpf, and then infected with *E*. *ictaluri* for 6 days. The numbers of surviving larvae were counted daily, and survival curves were generated. The number of *E*. *ictaluri* in larvae was calculated as the *in vivo* bacterial invasion assays. **p < 0.01.

The finding that overexpression of Bid could significantly inhibit the transcript of p53 with or without *E. ictaluri* infection (**Figure 6**) indicated that Bid exerts its antibacterial activity by regulating p53. Thus, we first analyzed the effects of p53 on the survival rates of fish infected with *E. ictaluri*. The survival rate of larvae microinjected with p53-Flag was significantly lower than that of fish microinjected with the p3×FLAG empty plasmid (*p* = 0.0002) (**Figure 7A**). We also found that the survival rates of larvae co-microinjected with Bid-FLAG and p53-FLAG were significantly increased (p < 0.01) compared with those of larvae microinjected with p53-Flag (**Figure 7A**). Furthermore, the *in vivo* bacterial invasion assays showed that the number of bacteria in larvae transfected with p53 was significantly higher than that of cells transfected with p3×FLAG empty plasmid (p < 0.01) and cells co-transfected with Bid-Flag and p53-FLAG (*p* < 0.01) (**Figure 7B**). Further, the number of bacteria in cells co-transfected with Bid-FLAG and p53-FLAG was significantly lower than that of cells transfected with p3×FLAG empty plasmid (*p* < 0.01) (**Figure 7B**).

### Antibacterial Activity of Bid Is Independent of Caspase-8

**Figure 8:**
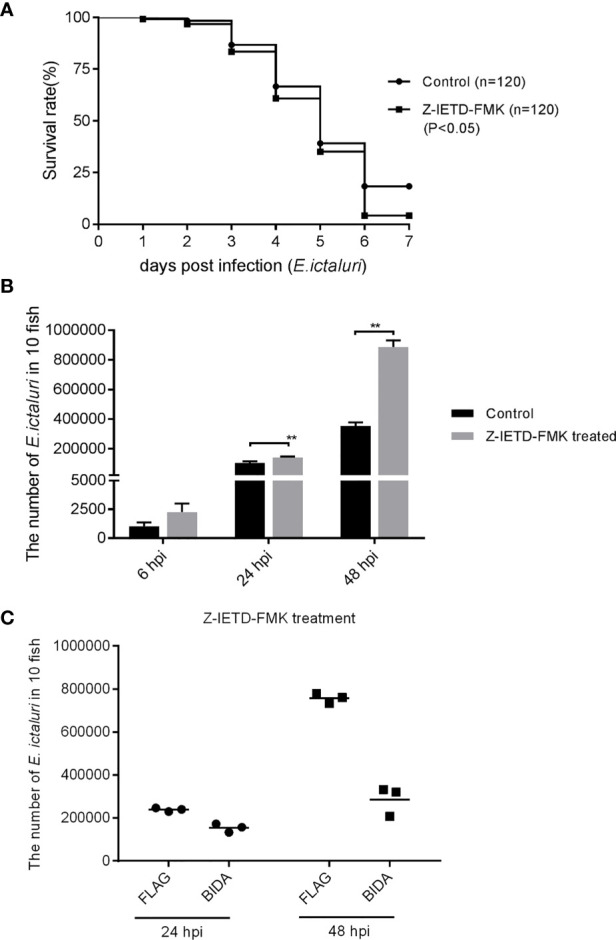
Antibacterial activity of Bid is caspase-8 independent. **(A)** Survival rates for larvae treated with Z-IETD-FMK. **(B)** Number of *E*. *ictaluri* in larvae treated with Z-IETD-FMK. **(C)** Number of *E*. *ictaluri* in larvae microinjected with Bid-Flag and treated with Z-IETD-FMK. **p < 0.01.

Z-IETD-FMK is the inhibitor of the caspase-8 inhibitor (15). We found that the larval survival at 6 dpi was decreased significantly when the larvae were pretreated with Z-IETD-FMK (**Figure 8A**). The *in vivo* bacterial invasion assays revealed that the numbers of *E. ictaluri* in larvae treated with Z-IETD-FMK at 24 and 48 hpi were higher than those of larvae without Z-IETD-FMK treatment (**Figure 8B**). Furthermore, we found that the number of *E. ictaluri* in larvae microinjected with *bid*-Flag decreased significantly, even if the larvae were treated in advance with Z-IETD-FMK (**Figure 8C**).

## Discussion

Bid, a member of the Bcl-2 family, plays a crucial role in mammalian apoptotic signaling (17). However, studies about its function in fish are scarce. In the present study, the *bid* of zebrafish was cloned, and its antibacterial function was investigated. Our results demonstrated that in addition to its pro-apoptotic function, zebrafish Bid has a strong antibacterial role against *E. ictaluri*, an important fish pathogen affecting cultured and wild fish worldwide (12, 18).

Zebrafish Bid encodes 187 amino acids, which share a high sequence identity with Bid of other fish species, ranging from 34.5% to 70.4% (**Figure 1**). Zebrafish Bid contains a conserved BH3 region that is required for Bid to bind to Bcl-2-like pro-survival proteins and initiate apoptosis (17). Phylogenetic tree analysis revealed that zebrafish Bid was well clustered with fish Bid (**Figure 2**), confirming that zebrafish Bid is a member of the Bid family. Phylogenetic tree analysis also showed that Bid forms from the different animal classes were separated, suggesting that Bid underwent divergent evolutionary processes.

Further analysis revealed some differences at the potential cleavage sites between zebrafish Bid and mammalian Bid (**Figure 1**). The caspase-8 cleavage site for mammalian Bid is “LQTD” (19), while the same site in zebrafish Bid is “IETD.” Also, mammalian Bid could be cleaved by granzyme B, which plays an important role in the target cell killing by cytotoxic T cells (20). The granzyme B cleavage site in mammalian Bid is “IEPD,” while that in zebrafish is “HELQ.” Mammalian Bid can also be cleaved by calpain, and the cleavage site is between Gly70 and Arg71 (21); a similar site in zebrafish is Leu67–Leu68. The poor conservation of these cleavage sites indicates that these enzymes might not be critical for the functions of zebrafish Bid.

*E. ictaluri* causes Edwardsiellosis in zebrafish (12). We found that following *E. ictaluri* infection, zebrafish *bid* was upregulated. Furthermore, zebrafish Bid could enhance the apoptosis rate of EPC cells following *E. ictaluri* infection (**Figure 3**). Our results are in line with the literature (1). A bid of Chinese giant salamander played a positive role in Chinese giant salamander iridovirus (GSIV)-induced apoptosis (22). Bid-deficient rare minnow delayed the grass carp reovirus (GCRV) replication and attenuated GCRV-induced apoptosis (10). Previous studies indicated that *Edwardsiella* could induce apoptosis in fish cells. For example, *E. tarda* (now renamed as *E. piscicida*) could induce apoptosis in EPC cells (14). These results indicated that Bid forms in lower vertebrates possess pro-apoptotic activity, similar to that of mammalian Bid.

We observed that the full length of zebrafish Bid has antibacterial activity against *E. ictaluri*, which could significantly inhibit the proliferation of *E. ictaluri*, as confirmed by the *in vitro* and *in vivo* bacterial invasion assays (**Figure 5**). To further understand the potential mechanism for Bid antibacterial activity, we checked the expression of potential genes related to Bid in larvae. As expected, we found that the overexpression of the full-length Bid could significantly upregulate the expressions of *birc5* and *egfr* in larvae infected with *E. ictaluri*. Birc5, also known as survivin, is an anti-apoptotic protein in vertebrates that can inhibit cell death (23). Egfr, a cell surface receptor, involves regulating cell growth in both normal and malignant tissues (24). The upregulation of these two genes indicated that they are involved in the cell apoptosis induced by Bid during *E. ictaluri* infection. Importantly, we observed that overexpression of the full-length Bid downregulated the expression of *p53* both in uninfected larvae and in larvae infected with *E. ictaluri* (**Figure 6**). The *p53* gene, an important transcription factor initially described as an oncogene, and later studies demonstrated it is part of the immune system (25). Downregulation of *p53* suggested that Bid might regulate p53 to exert antibacterial activity. Furthermore, we found that overexpression of *p53* could significantly decrease the survival rates of embryos following *E. ictaluri* infection. Further, the numbers of *E. ictaluri* in larvae were greatly increased, indicating that p53 plays a negative role during *E. ictaluri* infection. We also found that the survival rates of larvae co-microinjected with Bid-FLAG and p53-FLAG were significantly increased, and the numbers of *E. ictaluri* in EPC cells co-transfected with Bid-FLAG and p53-FLAG were decreased. Collectively, these results indicated that Bid could inhibit the negative role of p53 during *E. ictaluri* infection.

**Figure 9:**
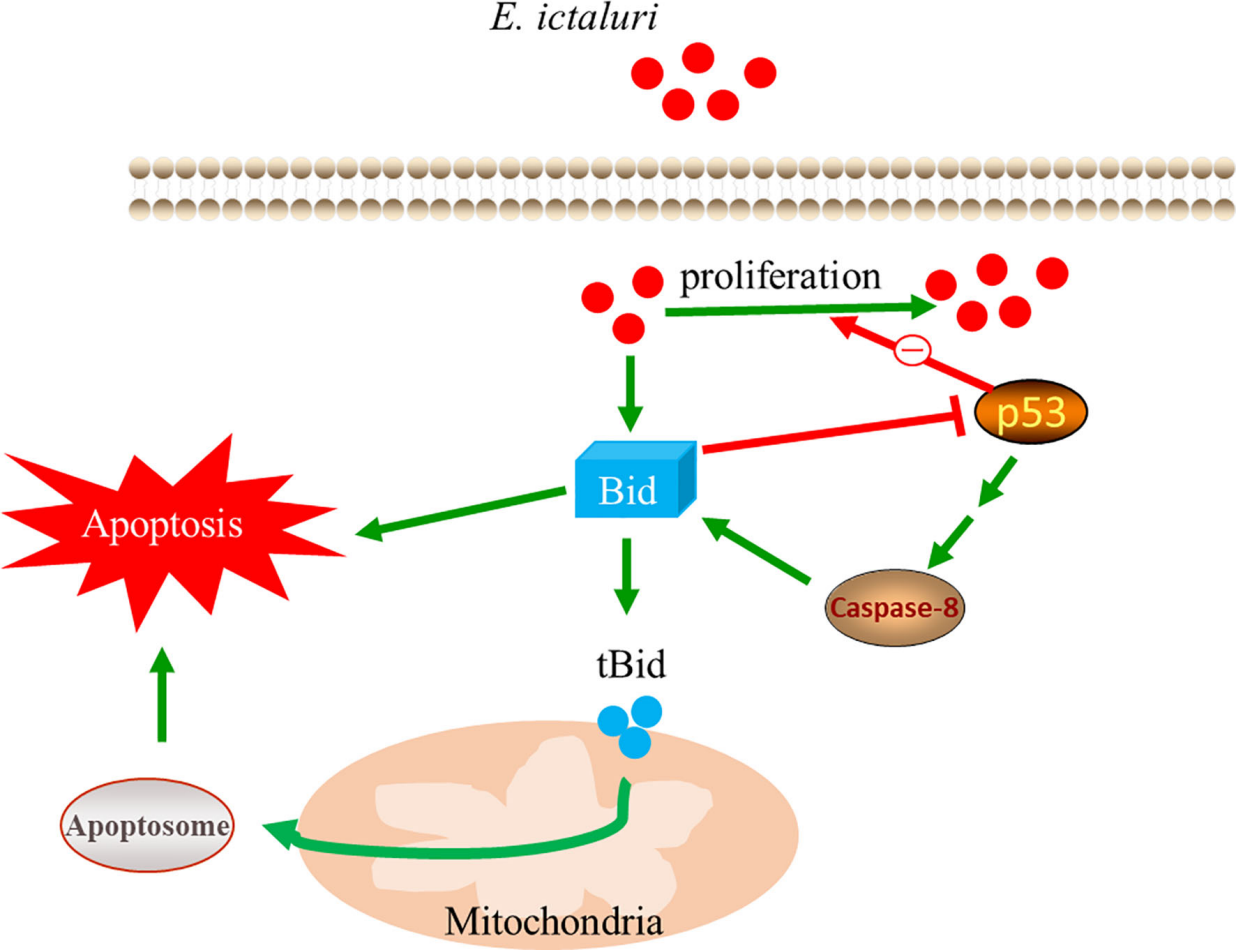
Mechanism of the antibacterial activity of full-length Bid.

Mammalian Bid needs to be cleaved by caspase-8 to exert most of its functions. However, some studies also found that functional Bid does not need to be cleaved by caspase-8. For example, a mutant Bid that lacks the caspase-8 cleavage site could still kill cells (26). Moreover, endogenous Bid could be translocated to the mitochondria without cleavage in an anoikis model (27). Here, we found that pretreatment of larvae with Z-IETD-FMK, an inhibitor of caspase-8, could significantly decrease the survival rate of larvae and increase the number of *E. ictaluri* in larvae, indicating that when caspase-8 was inhibited, the antibacterial properties were also inhibited. However, we found that despite the pretreatment of larvae with Z-IETD-FMK, the overexpression of Bid still had perfect antibacterial activity against *E. ictaluri*, indicating that the antibacterial activity of Bid does not rely on the cleavage of caspase-8. These results confirmed that the full-length Bid inhibit the p53 role to exert its antibacterial activity, which do not rely on the caspase-8-mediated Bid cleavage (**Figure 9**).

In summary, zebrafish Bid possess pro-apoptotic activity, similar to that of mammalian Bid. More importantly, zebrafish Bid had antibacterial activity, which could significantly inhibit the invasion and proliferation of *E. ictaluri in vivo* and *in vitro*. The antibacterial activity of Bid was achieved by inhibiting the negative role of p53 during *E. ictaluri* infection but does not rely on caspase-8-mediated Bid cleavage.

## Data Availability Statement

The datasets presented in this study can be found in online repositories. The names of the repository/repositories and accession number(s) can be found in the article/**Supplementary Material**.

## Ethics Statement

The animal study was reviewed and approved by the Animal Care and Use Committee of the Institute of Hydrobiology, CAS.

## Author Contributions

MC and ZQ designed the experiments and wrote the manuscript. DY performed the experiments. LC and YX helped in preparing the manuscript. All authors contributed to the article and approved the submitted version.

## Funding

This work was funded by the Strategic Priority Research Program of the Chinese Academy of Sciences (Grant XDA24010308) and the National Natural Science Foundation of China (Grant 31873046 and 31302221). ZQ was supported financially by the projects for “six talents” of Jiangsu Province (Grant No. NY-115).

## Conflict of Interest

The authors declare that the research was conducted in the absence of any commercial or financial relationships that could be construed as a potential conflict of interest.

## Publisher’s Note

All claims expressed in this article are solely those of the authors and do not necessarily represent those of their affiliated organizations, or those of the publisher, the editors and the reviewers. Any product that may be evaluated in this article, or claim that may be made by its manufacturer, is not guaranteed or endorsed by the publisher.
