# Supplementary material for: Zebrafish BID Exerts an Antibacterial Role by Negatively Regulating p53, but in a Caspase-8-Independent Manner
Source: Front Immunol. 2021 Aug 31;12:707426. doi: 10.3389/fimmu.2021.707426 (PMC8439435; doi:10.3389/fimmu.2021.707426)
Supplement: Supplementary file 1 [file Table_1.docx]

Table S1 Primers used in realtime qPCR analysis.

| Primer name | Sequence（5' to 3'） |
| --- | --- |
| GAPDHF | GTAACTCCGCAGAAAAGCCAGAC |
| GAPDHR | CAAAAGAAACTAACACACACACA |
| EF1αF | TTCTGTTACCTGGCAAAGGG |
| EF1αR | TTCAGTTTGTCCAACACCCA |
| TP53F | CTCAGGTTCCCGCAGTC |
| TP53R | TCCATTCAGCACCAAGC |
| CASP8LF | GTGTCTGTTGACGAAATACGA |
| CASP8LR | GTGACTGAATAAACCAGGAGC |
| BAXLF | CTCATCAGCACCGTTCAGT |
| BAXLR | TAGCGAGTTCTTCTCCAGTAA |
| BIDAF | CGTTCTGTCTCAAGCTGCTG |
| BIDAR | GCTGCCACATGCTGAAGTAA |
| BIRC5F | CGGAGGATGACCCTGAGAA |
| BIRC5R | AAGGACCACAGCCAAATGC |
| CASPASE3aF | TGTTCTTTATTCAGGCTTGTCG |
| CASPASE3aR | CTGCCATACTTTGTCATCATTT |
| CASPASE3bF | ACGGTGTAGGTGACGAGGAAAC |
| CASPASE3bR | AGGAGATAAACCAGGAGCCATT |
| EGFRF | GCATACGCACTGGAAACAAC |
| EGFRR | TGGGTCACAAGCCAAACAC |
| PDCD8F | TAAAGCCACAGCCAAGGACAC |
| PDCD8R | CACAGAACGATACCAACCACCA |
