# Supplementary material for: Zebrafish BID Exerts an Antibacterial Role by Negatively Regulating p53, but in a Caspase-8-Independent Manner
Source: Front Immunol. 2021 Aug 31;12:707426. doi: 10.3389/fimmu.2021.707426 (PMC8439435; doi:10.3389/fimmu.2021.707426)
Supplement: Supplementary file 2 [file Table_2.docx]

Table S2 The Ct values of the two reference genes (*gaphd* and *EF-1α*) used for qPCR.

|  |  | Ct (mean±SD) |
| --- | --- | --- |
| Without *E. ictaluri* infection | FLAG | 17.844±0.040 |
|  | Bid overexpression | 17.682±0.027 |
| With *E. ictaluri* infection | FLAG | 17.789±0.129 |
|  | Bid overexpression | 17.434±0.294 |
